# Feasibility of an Interactive Health Coaching Mobile App to Prevent Malnutrition and Muscle Loss in Esophageal Cancer Patients Receiving Neoadjuvant Concurrent Chemoradiotherapy: Prospective Pilot Study

**DOI:** 10.2196/28695

**Published:** 2021-08-27

**Authors:** Kyungmi Yang, Dongryul Oh, Jae Myoung Noh, Han Gyul Yoon, Jong-Mu Sun, Hong Kwan Kim, Jae Ill Zo, Young Mog Shim, Hyunyoung Ko, Jungeun Lee, Youngin Kim

**Affiliations:** 1 Department of Radiation Oncology Samsung Medical Center Sungkyunkwan University School of Medicine Seoul Republic of Korea; 2 Department of Medicine, Division of Hematology-Oncology Samsung Medical Center Sungkyunkwan University School of Medicine Seoul Republic of Korea; 3 Department of Thoracic and Cardiovascular Surgery Samsung Medical Center Sungkyunkwan University School of Medicine Seoul Republic of Korea; 4 Noom Korea Inc Seoul Republic of Korea; 5 Ingenium College of Liberal Arts KwangWoon University Seoul Republic of Korea; 6 Noom Inc New York, NY United States; 7 Department of Biomedical Systems Informatics Yonsei University College of Medicine Seoul Republic of Korea

**Keywords:** esophageal cancer, malnutrition, muscle loss, sarcopenia, mobile app, mHealth

## Abstract

**Background:**

Excessive muscle loss is an important prognostic factor in esophageal cancer patients undergoing neoadjuvant chemoradiotherapy (NACRT), as reported in our previous research.

**Objective:**

In this pilot study, we prospectively tested the feasibility of a health coaching mobile app for preventing malnutrition and muscle loss in this patient population.

**Methods:**

Between July 2019 and May 2020, we enrolled 38 male patients with esophageal cancer scheduled for NACRT. For 8 weeks from the start of radiotherapy (RT), the patients used Noom, a health coaching mobile app that interactively provided online advice about food intake, exercise, and weight changes. The skeletal muscle index (SMI) measured based on computed tomography and nutrition-related laboratory markers were assessed before and after RT. We evaluated the changes in the SMI, nutrition, and inflammatory factors between the patient group that used the mobile app (mHealth group) and our previous study cohort (usual care group). Additionally, we analyzed the factors associated with walk steps recorded in the app.

**Results:**

Two patients dropped out of the study (no app usage; treatment changed to a definitive aim). The use (or activation) of the app was noted in approximately 70% (25/36) of the patients until the end of the trial. Compared to the 1:2 matched usual care group by propensity scores balanced with their age, primary tumor location, tumor stage, pre-RT BMI, and pre-RT SMI level, 30 operable patients showed less aggravation of the prognostic nutritional index (PNI) (–6.7 vs –9.8; *P*=.04). However, there was no significant difference in the SMI change or the number of patients with excessive muscle loss (∆SMI/50 days >10%). In patients with excessive muscle loss, the walk steps significantly decreased in the last 4 weeks compared to those in the first 4 weeks. Age affected the absolute number of walk steps (*P*=.01), whereas pre-RT sarcopenia was related to the recovery of the reduced walk steps (*P*=.03).

**Conclusions:**

For esophageal cancer patients receiving NACRT, a health care mobile app helped nutritional self-care with less decrease in the PNI, although it did not prevent excessive muscle loss. An individualized care model with proper exercise as well as nutritional support may be required to reduce muscle loss and malnutrition.

## Introduction

Esophageal cancer is one of the most aggressive malignancies and is ranked as the sixth leading cause of cancer-related death worldwide [1]. Recent advances in treatment including radiotherapy (RT), chemotherapy, and surgery, and their combinations have led to improved clinical outcomes; however, patients continue to experience high mortality [2]. Patients with esophageal cancer commonly have symptoms such as dysphagia and weight loss even before a confirmed diagnosis of the disease, and up to 80% of patients are nutritionally compromised [3,4]. Malnutrition, cachexia, and sarcopenia are reported as poor prognostic factors associated with treatment compliance and clinical outcomes in esophageal cancer and various cancers other as well [5,6].

Previously, we conducted a retrospective review among 248 esophageal cancer patients who underwent surgery and reported excessive muscle loss after neoadjuvant concurrent chemoradiotheapy (NACRT) as a significant poor prognostic factor for disease recurrence and overall survival [7]. In that study, many patients experienced malnutrition before and after NACRT; 62.9% of the patients were assigned the status of sarcopenia before the start of the treatment, and 28.2% showed more than 10% deterioration in the skeletal muscle index (SMI) after NACRT. To reduce muscle loss, more active support toward the patient’s nutrition and physical activity was needed than education from a physician or nutrition specialist. Additionally, most patients were managed in the outpatient setting.

Noom (Noom Inc) [8] is one of the most popular commercial health care mobile apps. This app has been mainly used by obese people for weight control [9,10]. In addition to losing weight, the company also provides paramedical experience for patients with diabetes or eating disorders, and pregnant women [11-14]. It provides interactive health coaching on various aspects of nutrition, exercise, and weight control. Although there is little evidence for the usage of this app, it can be applied to the self-management of nutrition for cancer patients.

This prospective pilot study aimed to evaluate the usefulness of a health care mobile app in preventing malnutrition and excessive muscle loss in patients with esophageal cancer receiving NACRT.

## Methods

### Study Design

**Figure 1:**
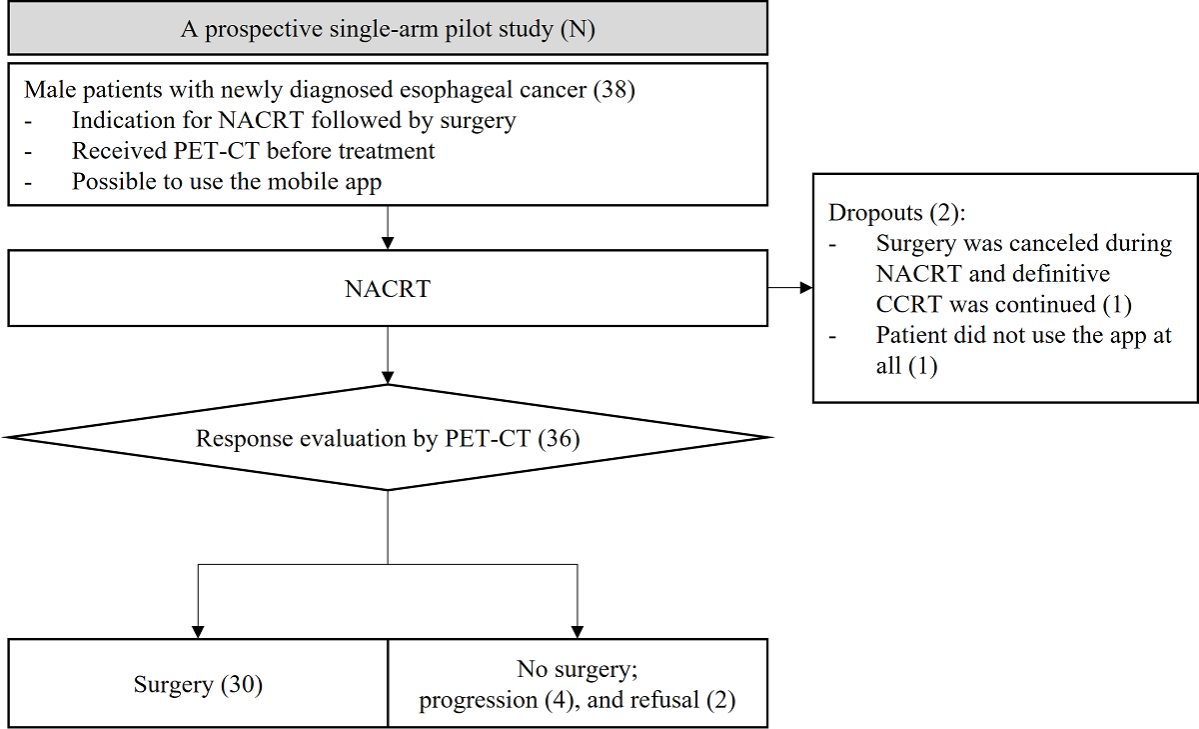
Flowchart of the study design. CCRT: concomitant radiotherapy and chemotherapy; NACRT: neoadjuvant chemoradiotherapy; PET-CT: positron emission tomography-computed tomography.

The inclusion criteria for this study were as follows: adult male patients (1) newly diagnosed with esophageal cancer, (2) scheduled for NACRT followed by radical surgery as per the authors’ institutional protocol, and (3) undergoing positron emission tomography-computed tomography (PET-CT) as part of the diagnostic workup (Figure 1). Patients with synchronous distant metastasis, previous history of thoracic irradiation, or having difficulty using an app on a smartphone were excluded. Between July 2019 and May 2020, totally 38 patients were enrolled in this study. Data regarding the app usage were gathered for 8 weeks from the beginning of NACRT. During the trial, two patients dropped out; one did not use the app at all, and the other decided not to undergo surgery during NACRT and continued RT with a definitive aim for 6 weeks. Finally, 36 patients analyzed to evaluate the feasibility and effectiveness of using the app. This study was approved by the Institutional Review Board of Samsung Medical Center, and all participants provided written informed consent.

### Treatment Scheme

Details of the treatment scheme, which includes RT and chemotherapy for NACRT, are described in our previous research [7]. The dose prescriptions for RT were 44 Gy in 22 fractions (2.0 Gy per fraction) for 18 patients and 43 Gy in 20 fractions (2.15 Gy per fraction) for 18 patients. All patients underwent intensity-modulated RT. All the patients completed NACRT as scheduled without grade 4 or higher complications. The concurrent chemotherapeutic regimen involved 2 cycles of cisplatin-based therapy in combination with 5-fluorouracil or capecitabine during RT. For response evaluation of NACRT, PET-CT was performed 3-4 weeks after the completion of NACRT, before surgery. The median time interval between pre-RT and post-RT PET-CTs was 73 days, ranging from 54 to 103 days. Patients underwent radical thoracic surgery, and the mean interval between the beginning of RT and completion of surgery was 10.5 weeks (range 9.1-14 weeks). Finally, surgery was not performed in 6 patients; 4 patients showed progressive disease after NACRT, not to assure curative surgery, and 2 patients did not want to undergo surgery after NACRT. Before NACRT, prophylactic feeding tube insertion was not performed in all patients except 1. During the trial, all patients received the best supportive care for their symptoms, such as painkillers and antacid drugs if needed.

### Mobile App for Health Coaching

Before the start of NACRT, patients were informed about the trial and app usage by a study instructor and attended an online meeting with the app manager. The app provided services following enrollment, and the patients used it autonomously. The app setting was similar to that used in previous studies [9]. During the first sign-in, patients set their information including age, sex, height, and current and target weight. Users could record their food intake, exercise, and weight on a daily basis. In addition, the activity monitor in the app automatically measured the number of walk steps. Based on these records, the app provided summaries on calorie balance and weight trends. Additionally, experts in nutrition and exercise therapy provided feedback messages and encouraged the users through the app. The number of active records and messages from patients was counted each week in terms of nutrition, weight, and exercise. If any active data were recorded by the patient in a certain week, then the app was considered “activated” for that week. Based on previous experiences of the app users, as the compliance for this trial, the activation level of the app per 8 weeks was categorized as follows: “high,” activated for 6-8 weeks; “moderate,” activated for 4-5 weeks; and “low,” activated for 1-3 weeks.

### Muscle Loss and Malnutrition Assessment

Similar to our previous study, the skeletal muscle area (cm^2^) at the level of the third lumbar vertebra (L3) in the two CT image sets from PET-CT before and after NACRT was measured using an in-house software program [15]. The SMI (cm^2^/m^2^) was calculated from the skeletal muscle area divided by the square of the height (m^2^). We also used the cutoff value of 52.4 cm^2^/m^2^ for sarcopenia from our previous study [7] and a population-based study [16]. ∆SMI (%), which is the percentage of SMI change based on the pre-RT SMI, was adjusted with various time intervals between 2 PET-CT measurements, and unified in 50 days per interval between 2 PET-CT measurements for each patient (∆SMI/50 days, %). Excessive muscle loss was defined when ∆SMI/50 days was more than 10%.

Other information regarding nutritional status was obtained from the laboratory tests on the day closest to that before the start of RT (pre-RT) and those as preoperative workups (post-RT), which were the first blood tests in most patients performed after the end of RT. Data were collected for the following laboratory parameters: absolute counts of white blood cells (WBC, /μL), absolute neutrophil count (ANC, /μL), absolute lymphocyte count (ALC, /μL), platelet count (/μL), and albumin level (g/dL). From these results, the neutrophil-to-lymphocyte ratio (NLR, ANC/ALC), platelet-to-lymphocyte ratio (platelet/ALC), and prognostic nutritional index (PNI, 10 × albumin + 0.005 × ALC) [17] were calculated.

### Statistical Analyses

The purpose of this study was to evaluate whether ∆SMI (%/50 days) was reduced in patients who used the mobile app (mHealth group) compared with that in our previous study cohort (usual care group). Additionally, changes in nutritional and inflammatory factors were also analyzed between the two groups. To compare the two groups, propensity score matching was used to balance characteristics such as the age, primary tumor location, tumor stage, pre-RT BMI, and pre-RT SMI level. Fisher tests for discrete variables and *t* tests (or Mann-Whitney *U* test) for continuous variables were conducted for comparing the characteristics. To identify significant factors associated with excessive muscle loss in this study, logistic regression was performed with the quantified app data. Additionally, we performed mixed model analysis for repeated measures of walk steps counted by the app for 8 weeks. R 4.0.3 (version 4.0.3, R Development Core Team) [18] and SPSS Statistics (version 27.0, IBM Corp) were used for statistical analyses with *P*<.05 suggesting statistical significance.

## Results

### Compliance With the App

For 8 weeks of the trial, 80.6% (29/36), 77.8% (28/36), 75.0% (27/36), 77.8% (28/36), 72.2% (26/36), 66.7% (24/36), and 72.2% (26/36) of the patients had been active on the app from the second week to the 8th week. In addition, the activation levels were as follows: high, moderate, and low levels in 25 (69.4%), 3 (8.3%), and 8 (22.2%) patients, respectively. However, the patients selectively used parts of the app (Table 1). The most activated records were for the nutritional part. However, in the exercise records, only one was moderately activated, and there was no highly activated patient. Walk steps were passively gathered and had a pattern similar to that of the total activation level.

**Table 1:** Activation level based on the number of activated weeks.

Activated part of the app	Activation level^a^, n (%) (N=36)		
	Low	Moderate	High
At least one activation in any part of the app	8 (22.2)	3 (8.3)	25 (69.4)
Nutrition	14 (38.8)	7 (19.4)	15 (41.7)
Weight	20 55.6)	4 (11.1)	12 (33.3)
Exercise	35 (97.2)	1 (2.8)	0 (0)
Walking^b^	6 (16.7)	5 (13.9)	25 (69.4)

^a^Activation level was determined depending on the number of activated weeks—low: activated for 1 to 3 weeks; moderate: activated for 4 to 5 weeks; high: activated for 6 to 8 weeks.

^b^The number of walk steps was automatically recorded by the activated app.

### Patient Characteristics and Comparison With the Usual Care Group

The median patient age was 59 years, and all patients had an Eastern Cooperative Oncology Group Performance Status (ECOG PS) of 0 to 1. The details of the patient characteristics are summarized in Table 2. Compared to the previous study cohort (usual care group), the age, tumor location, and some of the laboratory markers were significantly different in this study cohort (mHealth group) (Supplement table). As a result, ∆SMI/50 days (%) and the proportion of patients with excessive muscle loss (∆SMI/50 days >10%) were not significantly different between the two groups. However, in the mHealth group, the PNI decreased to a lesser extent after NACRT.

After 1:2 propensity score matching to adjust for variables such as the age, primary tumor location, tumor stage, pre-RT BMI, and pre-RT SMI level, we compared 30 patients in the mHealth group with those of the usual care group (Table 2). Even after propensity score matching, ∆SMI/50 days (%) and the proportion of patients with excessive muscle loss (∆SMI/50 days >10%) were not significantly different (–8.1% vs 7.4%, *P*=.57 and 33.3% vs 30.0%, *P*=.94, respectively). The PNI decreased to a lesser extent in the mHealth group than in the usual care group (–6.7 vs –9.8, *P=.*04).

**Table 2:** Propensity score matching and comparison in 30 patients who underwent surgery after neoadjuvant concurrent chemoradiotherapy.

Characteristics				Previous cohort [7] (n=60)		mHealth group (n=30)^a^		*P* value	
Age (years), median (SD)				58.5 (7.8)		59.2 (6.5)		.8	
**Age group, n (%)**								.99	
	<60 years		34 (56.7)		17 (56.7)				
	≥60 years		26 (43.3)		13 (43.3)				
**Smoking status, n (%)**								.41	
	Current smoker		35 (58.3)		14 (46.7)				
	Ex- or nonsmoker		25 (41.7)		16 (53.3)				
**Tumor location, n (%)**								.73	
	Upper		12 (20)		4 (13.3)				
	Middle		21 (35)		11 (36.7)				
	Lower		27 (45)		15 (50)				
**cT stage^b^, n (%)**								.49	
	1-2		24 (40)		9 (30)				
	3-4		36 (60)		21 (70)				
**cN stage^c^, n (%)**								.94	
	0-1		34 (56.7)		18 (60)				
	2-3		26 (43.3)		12 (40)				
Pre-RT^d^ BMI (kg/m^2^), mean (SD)				22.9 (2.8)		22.9 (2.3)		.99	
**Pre-RT BMI (kg/m^2^)**								.99	
	<20, n (%)		7 (11.7)		3 (10)				
	≥20, n (%)		53 (88.3)		27 (90)				
Post-RT BMI (kg/m^2^), mean (SD)				22 (6)		21.8 (2.6)		.65	
**Post-RT BMI (kg/m^2^)**									
	<20, n (%)		10 (16.7)		7 (23.3)		.63		
	≥20, n (%)		50 (83.3)		23 (76.7)				
∆BMI (kg/m^2^), mean (SD)				-0.8 (1.4)		-1.1 (1.2)		.38	
Pre-RT SMI^e^ (cm^2^/m^2^), mean (SD)				51.0 (9.1)		51.3 (6.2)		.87	
**Pre-RT SMI** **(cm^2^/m^2^)**								.99	
	Sarcopenia, n (%)		36 (60)		18 (60)				
	Nonsarcopenia, n (%)		24 (40)		12 (40)				
Post-RT SMI (cm^2^/m^2^), mean (SD)				45 (7.8)		45.9 (6.9)		.58	
**Post-RT SMI (cm^2^/m^2^)**								.93	
	Sarcopenia, n (%)		48 (80)		23 (76.7)				
	Nonsarcopenia, n (%)		12 (20)		7 (23.3)				
ΔSMI (/50 days), mean, % (SD)				–8.1 (5.3)		–7.4 (6.5)		.57	
**ΔSMI (/50 days)**								.94	
	Decreased >10%, n (%)		20 (33.3)		9 (30)				
	Decreased <10%, n (%)		40 (66.7)		21 (70)				
**Laboratory markers**									
	**WBC^f^(×10^3^/μL), mean (SD)**								
		Pre-RT	8.2 (2.5)		7.1 (1.7)		.02		
		Post-RT	4.8 (2.2)		4.7 (2.8)		.93		
		Δ	–3.4 (3.5)		-2.3 (2.8)		.05		
	**ANC^g^(×10^3^/μL), mean (SD)**								
		Pre-RT	5.1 (2.2)		4.4 (1.3)		.2		
		Post-RT	2.8 (2)		2.6 (1.6)		.6		
		Δ	­–2.4 (3.1)		–1.8 (2.1)		.34		
	**ALC^h^(×10^3^/μL), mean (SD)**								
		Pre-RT	2.2 (0.5)		1.9 (0.6)		.02		
		Post-RT	1.3 (0.6)		1.5 (1.3)		.6		
		Δ	–0.9 (0.7)		–0.4 (1.3)		.11		
	**Platelet(×10^3^/μL), mean (SD)**								
		Pre-RT	263.0 (72.2)		260.3 (52.9)		.86		
		Post-RT	215.0 (70.3)		203.5 (74)		.48		
		Δ	–48.0 (69.3)		–56.7 (84.2)		.6		
	**Albumin** **(g/dL),** **mean (SD)**								
		Pre-RT	4.4 (0.3)		4.4 (0.3)		.19		
		Post-RT	3.8 (0.5)		4.0 (0.4)		.07		
		Δ	–0.6 (0.4)		–0.5 (0.4)		.34		
	**NLR^i^, >mean (SD)**								
		Pre-RT	2.4 (1.0)		2.5 (1)		.77		
		Post-RT	2.8 (3.7)		3.1 (4.7)		.75		
		Δ	0.4 (3.9)		0.6 (5.1)		.82		
	**PLR^j^, mean (SD)**								
		Pre-RT	125.3 ( 41.8)		149.8 (54.1)		.02		
		Post-RT	209.4 (159.6)		212.1 (160.8)		.94		
		Δ	84.1 (157.6)		62.4 (173.4)		.55		
	**PNI^k^, mean (SD)**								
		Pre-RT	54.6 (4.2)		54 (4.2)		.54		
		Post-RT	44.8 (5.7)		47.3 (7.7)		.09		
		Δ	–9.8 (6)		–6.7 (7.5)		.04		

^a^Patients who received surgery.

^b^cT: clinical tumor stage.

^c^cN: clinical nodal stage.

^d^RT: radiotherapy.

^e^SMI: skeletal muscle index.

^f^WBC: white blood cells.

^g^ANC: absolute neutrophil count.

^h^ALC: absolute lymphocyte count.

^i^NLR: neutrophil-to-lymphocyte ratio.

^j^PLR: platelet-to-lymphocyte ratio.

^k^PNI: prognostic nutritional index.

### Use of the App and the Associated Excessive Muscle Loss

In this study, there was excessive muscle loss in 12 of the 36 patients (33.3%) (Table 3). Regarding the app usage, there was no significant difference in the activation level, but walk steps decreased more in the excessive muscle loss group. More patients with a 70% or more decrease in walk steps between the first and the last 4 weeks were in the excessive muscle loss group (66.7% (6/9) vs 14.3% (3/21), *P=.*02).

**Table 3:** App usage and excessive muscle loss.

App usage		Nonexcessive muscle loss group (n=24)	Excessive muscle loss group (n=12)	*P* value	
**App activation level, n (%)**					.96
	Low	5 (20.8)	3 (25)		
	Moderate	2 (8.3)	1 (8.3)		
	High	17 (70.8)	8 (66.7)		
**First to fourth week, n (%)**					.99
	No full activation	6 (25)	3 (25)		
	Full activation	18 (75)	9 (75)		
**Fifth to eighth week, n (%)**					.11
	No full activation	6 (25)	7 (58.3)		
	Full activation	18 (75)	5 (41.7)		
**Walk steps^a^**					
	Steps per week/10^3^, mean (SD)	15.8 (13.1)	16.8 (17)	.85	
	First to fourth week (a), mean (SD)	16.9 (13.2)	18.4 (18.2)	.79	
	Fifth to eighth week (b), mean (SD)	10.4 (10.5)	5.6 (9)	.22	
	Δ (b–a, %), mean (SD)	–33.1 (64.4)	–69.0 (35.5)	.13	
	Decrease <70%, n (%)	18 (85.7)	3 (33.3)	.02	
	Decrease >70%, n (%)	3 (14.3)	6 (66.7)	—^b^	

^a^Nonexcessive muscle loss group (n=21); excessive muscle loss group (n=9).

^b^Not applicable.

### Mixed Model Analysis for Weekly Walk Steps

**Figure 2:**
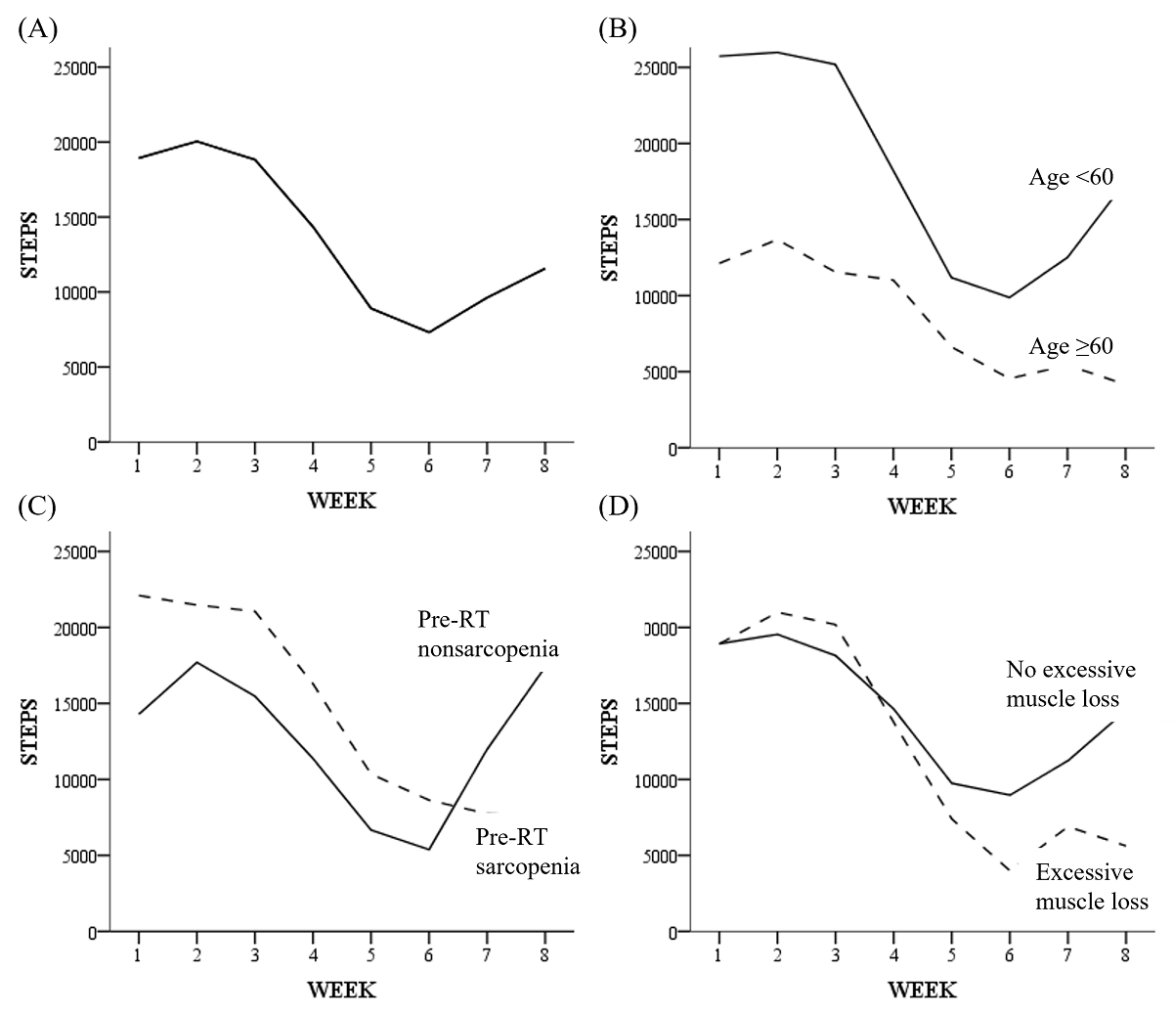
Weekly trends in walk steps recorded automatically by the application for 8 weeks (N=33): all patients (A), patient groups according to age (B), pre-RT sarcopenia (C), and excessive muscle loss (-ΔSMI/50 days >10%), during neoadjuvant concurrent chemoradiotherapy. RT: radiotherapy; SMI: skeletal muscle index.

Repeatedly measured walk steps appeared to have a pattern (Figure 2A), which decreased at the 5th and 6th weeks and slightly recovered at the 7th and 8th weeks. Discrete variables such as age, BMI, SMI, and surgery were used for this analysis (Table 4). When the variables were fixed, time (week) was commonly significant. In contrast, when time was fixed, only the age was significantly different (*P=.*01), which showed a gap in the absolute number of walk steps in the two age groups (Figure 2B). For the interaction between time and each variable, only pre-RT sarcopenia was significantly different (*P=.*03), which showed different recovery patterns at the 6th and 8th weeks between the pre-RT sarcopenia and nonsarcopenia groups (Figure 2C). Although not significant, excessive muscle loss seemed to affect the trend in the number of walk steps (Figure 2D).

**Table 4:** Mixed model analysis with walk steps for 8 weeks.

For 8 weeks	*P* value for weeks	*P* value for variables	*P* value for interaction
Age (<60 vs ≥60 years)	<.001	*.006*	.39
Pre-RT^a^ BMI (≥20 vs <20 kg/m^2^)	.001	.17	.98
Post-RT BMI (≥20 vs <20 kg/m^2^)	.001	.67	.47
-ΔBMI (>5% vs <5% kg/m^2^)	<.001	.98	.096
Pre-RT sarcopenia (yes vs no)	<.001	.55	.03
Post-RT sarcopenia (yes vs no)	.001	.44	.64
-ΔSMI^b^/50 days >10% (>10% vs <10%)	<.001	.42	.43
Surgery (Yes vs No)	.007	.08	.96

^a^RT: radiotherapy.

^b^SMI: skeletal muscle index.

## Discussion

### Principal Findings

Sarcopenia is a syndrome characterized by a progressive decrease in skeletal muscle mass and strength, leading to a risk of worse outcomes in terms of physical ability, quality of life, and survival, although there are various definitions for this term provided by the European Working Group on Sarcopenia in Older People (EWGSOP), the European Society for Clinical Nutrition and Metabolism Special Interest Groups (ESPEN-SIG), and the International Working Group on Sarcopenia (IWGS) [19]. It was first considered an age-related disease by Rosenberg [20]. However, in recent years, it is clinically noticeable that sarcopenia can be caused by malignancy or inflammatory diseases and adversely affects their treatment. The mechanism of sarcopenia is not clear but seems multifactorial; its risk factors are old age; female sex; low level of physical activity; and comorbidities including malignancy, obesity, osteoporosis, and diabetes [21]. Focusing on malignancy, higher metabolism, and inflammation by aggressive cancer cells, cancer treatments including surgery, chemotherapy, and RT; anorexia or poor oral intake; and low physical activity make patients more susceptible to sarcopenia [22]. Moreover, gastrointestinal malignancy easily causes malnutrition, and most esophageal cancer patients experience cachexia or sarcopenia. Furthermore, the presence of sarcopenia in cancer patients has been accepted clinically as one of the important predictors of survival and treatment outcomes [6,22]. In addition to clinical evidence, the muscle loss is gaining interest in biochemical research, including the anti-inflammatory or anticancer effects of myokines [23]. We previously analyzed the effect of sarcopenia in patients with esophageal cancer receiving NACRT [7]. NACRT itself has risk factors for sarcopenia; symptoms or complications such as nausea/vomiting and anorexia induced by chemotherapy and acute esophagitis by RT can deplete nutrition and physical energy [22]. We revealed that the changes in the muscle index (∆SMI/50 days) were the prognostic factors for disease recurrence and survival and not the presence of sarcopenia before the treatment [7]. Additionally, sarcopenia is related to nutritional and inflammatory markers [7,24]. Therefore, we suggest that appropriate intervention for nutrition and physical activity could be beneficial for these patients.

Numerous interventions to overcome malnutrition in patients with various cancers have been attempted [3,25,26]. Unfortunately, interventions to improve cancer patients’ malnutrition or muscle loss have been rare. Malnutrition is still considered insurmountable, and most esophageal cancer patients find it difficult to overcome malnutrition or often experience worsened conditions. This study, which used an interactive health coaching mobile app as an intervention for nutrition and physical activity, found similar negative results in terms of preventing muscle loss in esophageal cancer patients receiving NACRT compared to that in the usual care group. As a secondary endpoint, however, the use of the mobile app could improve nutritional indicators such as PNI, which is also known to be a prognostic factor for gastrointestinal cancers [17]. The compliance of the mobile app was as good because 70% (20/36) of the patients used it until the end of the trial, especially in nutrition and weight items. This could be because the patients were concerned about maintaining their oral intake and weight.

Guidelines for nutritional support suggest that appropriate interventions have to be selected depending on each patient’s nutritional status, from basic nutritional modification and oral nutritional supplements to enteral or parenteral nutrition for severe cases [3,5]. However, it might be difficult by only through nutritional interventions to improve or prevent sarcopenia. The benefit of physical activity and exercise is well known for sarcopenia, and the international clinical practice guidelines for sarcopenia in 2018 strongly recommend physical activity for the treatment of sarcopenia [27]. Any single intervention in the aspect of nutrition, exercise, or medication is less effective for the management of sarcopenia, and proper dietary intake and exercise should be combined [28]. Resistance exercise has been shown to improve muscle mass and strength [29,30].

An interesting result was the number of weekly walk steps. The mean of the walk steps reached a maximum of 20,000 in the second week and a minimum of 7000 in the 6th week. It is assumed that the limited physical activity during the fourth to the 6th week could cause muscle loss. This pattern is associated with the severity of radiation esophagitis. In general, RT esophagitis begins at week 2 of RT, its severity increases over time, and peaks at the end of RT. It takes approximately 3 weeks for the symptoms to resolve. Detailed analyses of the walking steps showed that patients with excessive muscle loss during NACRT significantly decreased the number of steps in the last half of the trial period. Furthermore, age affected the number of walking steps, and pre-RT sarcopenia showed a difference in the recovery of the decreased walking steps after NACRT. This means that older patients and those with sarcopenia at the start of the treatment have difficulty maintaining physical activity during the treatment and recovering decreased physical activity after NACRT. Some studies suggested that the number of walking steps was related to performance status [31]. As performance status is also one of the important factors for surgical indication and outcome [32], the pattern of the decrease and recovery in walking steps after NACRT is likely to be an indicator for the continuation of follow-up surgery or a predictor for postoperative mortality in patients with esophageal cancer.

To prevent malnutrition and muscle loss in esophageal cancer patients receiving NACRT, comprehensive supportive care including nutritional care as well as exercise might be needed. In addition, the status of nutrition and muscle mass needs to be evaluated individually before the start of treatment. For example, patients without malnutrition and sarcopenia can be managed with the mobile interactive coaching app used in this study. However, patients with old age, malnutrition, and sarcopenia might need to be managed differently. Physical activity guidelines for cancer patients from the American Cancer Society recommend avoiding inactivity, continuing normal daily activity as much as possible, and performing exercise adapted for the disease and the patients’ condition, particularly if patients experience severe symptoms such as extreme fatigue or ataxia, or have cardiovascular and pulmonary contraindications [33]. However, exercise programs targeting patients with sarcopenia or extremely low physical activity are not well established, and high-intensity exercise might be not good for patients undergoing cancer treatment. It is necessary to develop a customized and intensity-modified physical program for patients with malnutrition and sarcopenia.

As a limitation, this prospective study was a small pilot test, thus limiting detailed analyses. We used the SMI from CT to diagnose sarcopenia, but muscle strength, which is one of the criteria for the definition of sarcopenia, could not be evaluated. In addition, all enrolled patients were male for comparison with our previous study, which predominantly involved esophageal cancer patients for ensuring study homogeneity. Finally, we focused on the app usage such as the activation or automatically recorded walk steps, but more specific nutritional factors such as calories during oral intake were missing. Although the app was not highly effective in preventing muscle loss and further research using more detailed information is needed, we expect that it can be used as a self-managing nutritional support for cancer patients and possibly expanded for cancer survivors after treatment.

### Conclusion

For esophageal cancer patients receiving NACRT, an interactive health coaching mobile app helped nutritional self-care with a significantly less decrease in PNI, although it did not prevent excessive muscle loss. Low physical activity estimated by the number of walking steps did not recover even a few weeks after the end of NACRT in patients with old age or pretreatment sarcopenia. An individualized care model with proper exercise as well as nutritional support may be required to reduce muscle loss and malnutrition.

## Abbreviations

ALC: absolute lymphocyte count
ANC: absolute neutrophil count
ESOG PS: Eastern Cooperative Oncology Group Performance Status
ESPEN-SIG: European Society for Clinical Nutrition and Metabolism Special Interest Groups IWGS: International Working Group on Sarcopenia
EWGSOP: European Working Group on Sarcopenia in Older People
NACRT: neoadjuvant chemoradiotherapy
NLR: neutrophil-to-lymphocyte ratio
PNI: prognostic nutritional index
RT: radiotherapy
SMI: skeletal muscle index
WBC: white blood cell
